# Preparedness for treating injured patients at a single-centre trauma hospital in Ethiopia: a qualitative study

**DOI:** 10.1080/16549716.2025.2540669

**Published:** 2025-08-05

**Authors:** Helina Bogale Abayneh, Stine Engebretsen, Kristin Halvorsen, Stein Ove Danielsen

**Affiliations:** a Department of Nursing and Health Promotion, Faculty of Health Sciences, Oslo Metropolitan University, Oslo, Norway; b Department of Emergency and Critical Care Nursing, St Paul Hospital Millennium Medical College, Addis Ababa, Ethiopia; c Emergency Department, Oslo University Hospital, Oslo, Norway

**Keywords:** Trauma care, readiness, limited resource, low-income country, quality of care

## Abstract

**Background:**

Across the world, a disproportionate amount (90%) of injury-related deaths and disabilities occur in Global South countries where one-third of injury deaths could be prevented. In most Global South countries, there is varying availability of resources, and many hospitals lack important equipment, some of which is inexpensive.

**Objective:**

To explore the perceptions of the staff regarding the preparedness of handling traumatic injuries at Addis Ababa Burn Emergency and Trauma Hospital in Ethiopia.

**Method:**

A qualitative, exploratory study was conducted using semi-structured interviews. The sample pool included a diverse group of professionals who work in various roles within the hospital where the interview and data generation were conducted in early 2023. The interviews were analyzed using the reflexive thematic analysis approach outlined by Braun and Clarke.

**Results:**

Resident doctors, who bear the primary responsibility for patient care, often face delays in decision-making, which contribute to procedural backlogs and extended patient stays. A significant challenge was identified in the availability, maintenance, and procurement of medical equipment, alongside shortages and inconsistencies in the supply of medicines and materials. A notable knowledge and skill gap among healthcare professionals was observed, compounded by the absence of a standardized trauma response team. Furthermore, the interview clarifies that there is a substantial gap and significant challenges in quality of service and standardization.

**Conclusions:**

There are significant gaps in terms of hospital preparedness for caring for injured patients, and these lacks can lead to delays in treatment and poor clinical outcomes.

## Paper context

- **Main findings**: Gaps in hospital preparedness, delays in decision-making, equipment shortages, inconsistent supplies, and lack of structured trauma protocols hinder effective patient care.
- **Added knowledge**: The dedication and goodwill of healthcare professionals are vital in delivering quality care despite limited resources, emphasizing the need to use available assets effectively.
- **Global health impact for policy and action**: It is important to implement structured protocols, establish dedicated trauma teams, conduct regular training, and improve equipment and supply management systems to enhance patient care.

## Background

Each year, trauma causes more deaths worldwide than the combined toll of HIV/AIDS, malaria, and tuberculosis [1]. For every trauma death, there are 20–50 nonfatal injuries that result in some form of disability, impacting quality of life, productivity, and financial security [2].

Across the world, a disproportionate amount (90%) of trauma-related deaths and disabilities occur in Global South countries [3]. In 2019, Ethiopia, Uganda, Zimbabwe, and South Africa had the highest proportions of death by injury in the region [4]. According to the United Nations Economic Commission, the region most affected by road injuries is sub-Saharan Africa, with a fatality rate of 27 per 100,000 inhabitants. This is three times higher than Europe’s average of nine and well above the global average of 18 [5].

It is well known that Global South countries have lower survival rates for equivalent injuries compared to high-income countries. Every year, one-third of trauma-related deaths could be prevented through improved trauma care provision for the injured, as evidenced by the successful implementation of trauma care systems in high-income countries, which has led to a significant reduction in both mortality and disability [6]. In most Global South countries, there is varying availability of resources, and little consideration has been given to optimizing the preparation of medical and nursing staff for the care of injured patients. Many hospitals lack important equipment, some of which is inexpensive [7].

Although prevention is essential and there are many effective and cost-effective injury prevention strategies [3], no system will prevent all injuries [8]. Robust evidence indicates that well-organized trauma care can save lives once an injury has occurred [9]. In addition to the higher trauma burden experienced in Global South countries, including a high injury rate, a younger age group of victims, and significant costs, these countries encounter various injury care limitations. These constraints primarily revolve around resource scarcity, insufficient availability of well-trained personnel, and a lack of adequately equipped health-care facilities, particularly within hospital settings [10]. Furthermore, there is a growing unmet need for post-injury rehabilitation services, including physical and occupational therapy. In Global South countries, more than half of those in need do not have access to such care [11].

Measuring the quality of care provided after a traumatic event serves as an ideal indicator and a valuable marker of the strength of the injury care system [12]. Gaining a comprehensive understanding of preparedness within a trauma center is crucial for effective trauma system management. However, there is still limited data available regarding access to quality healthcare services for the injured in Global South countries, highlighting the need to prioritize research in this area [13].

Analysis based on the global burden of disease study report indicates that injuries pose an escalating public health issue in Ethiopia. The age-standardized death rate due to injuries remains high, and the current annual reduction falls short of expectations [14]. This study therefore aimed to explore the preparedness and readiness of a hospital, located in Addis Ababa, Ethiopia, in providing care for traumatic and injured patients. The findings of this study will provide valuable insights that can guide the enhancement of essential structural elements within the center and possibly to other hospitals. Ultimately, the results of this study could be used to improve care for patients in need of trauma care.

## Methodology

### Study design

A qualitative, exploratory study was conducted, using semi-structured interviews to explore the perceptions and experiences of the hospital staff regarding the preparedness of handling traumatic injuries.

### Study context

The hospital has a trauma and emergency center offering 24/7 emergency services. Although the emergency room has dedicated personnel, there is currently no established or standardized trauma team to provide care for severely injured patients who require multi-disciplinary medical services or procedures. Instead, the care team is assembled after the first consultation. Upon arrival, the triage team conducts an initial assessment, and then assigns the patient to either a treatment zone or the waiting area based on the severity of their conditions. The emergency department (ED) has three treatment/resuscitation areas based on the triage scale: The Red area with 5 beds, the Orange with 8 beds, and the Yellow and Green with 30 recliners. The South African Triage System [15] with five triage levels is currently in use.

### Recruitment strategies

To ensure that the study participants had sufficient knowledge and experience related to the care of trauma patients, a strategic sampling strategy was employed. The sample pool included a diverse group of professionals who work in various roles and departments within the healthcare system. These included registered nurses and physicians, support and administrative staff. All of these individuals had experience in caring for traumatic and injured patients as well as in managing the care. Ultimately, the strategic sample consisted of 10 participants to ensure variation and sufficient information power from the sample and consideration of saturation [16,17].

### Dataset generation

The dataset generation was conducted in early 2023. The interviews were conducted by the first author (HB), who is an Emergency Medicine and Critical Care Nurse specialist with sparse experience in practical qualitative research. The interview guide tool (supplement 1) was carefully prepared by the authors based on the research objective and the types of the participants to ensure the questions were relevant, comprehensive, and aligned with the study’s goals. Prior to data generation, the interview guide was pre-tested among three nurses to improve question flow and structure, identify any gaps or inconsistency and to assess the time and length to ensure an appropriate reliability in the coming conversations with the participants. This was also the first planned research interviews by the first author, thus appropriate and valuable training for HB. The team with HB also provided guidance before the interviews took place. The individual interview approach was chosen as the preferred method to gain an in-depth understanding as well as maximum variation on the topic. During the interviews, HB strived to and ensured that the participants were given the space to express their insights in a manner that best reflected their perceptions and experiences. The semi-structured interview began with open-ended questions, followed by subsequent questions based on the participants’ answers, creating a dynamic conversation.

The first author provided detailed information to the participants about their privacy rights, and the participants gave their oral and written consent, and agreed to have their interviews audio recorded. The date and time for the interviews were arranged based on the participants’ preferences. Each interview-time ranged from 20 to 45 minutes. All interviews were conducted in Amharic, which is the national language fluently spoken by the participants.

### Data analysis

The first author is also affiliated with the hospital where the participants were recruited from in this study. For this reason, she made a concerted effort to adjust for the potential biases during the interviews and analysis. The first author listened to the audio recordings and transcribed the interviews. The full text of the interviews was then translated from Amharic to English. The interviews were analyzed using the reflexive thematic analysis approach, involving six phases following the method outlined by Braun and Clarke [18]: familiarization, generating codes, constructing themes, revising (reviewing) themes, defining and naming themes, and producing the report. Thematic analysis allows researchers to act as active participants in the analysis process. It helps us provide deep insights into participants’ experiences, while the analysis process is iterative and recursive. This means we move back and forth between stages of data familiarization, coding, and theme development, leading to robust and meaningful findings based on our interpretations.

The first author employed a rigorous approach to the analysis, with both a deductive and inductive approach. By transcribing the text in both Amharic and English gave HB a deep understanding that supported the generation of initial themes as a starting point. During this familiarization phase of the analysis, HB read and re-read the transcripts to further identify information related to the potential themes of interest and gain even more familiarity with the data. The analysis content was shaped and constructed through all phases in several analysis panels that were held with SOD, SE, and KH together with HB. This gave strength to the reflexivity of the data provided by the participants.

Codes were determined, analyzing the text line by line and continuously appraising the data across form the different interviews. At the initial stages of the analysis, HB consistently compared codes within each interview to look for similarities and differences. Whenever a new idea about what information was relevant and distinct different from the existing codes, new codes were determined. This coding process persisted until all the transcripts were thoroughly analyzed.

Following the identification of relevant codes, they were sorted, organized, and merged according to their similarities and differences to create sub-themes. By grouping and combining the codes, a total of four sub-themes were established, which were then assigned to overarching categories that captured the essence and substance of the codes. Throughout this process, the coherence, consistency, and relevance of the codes within each potential sub-theme were taken into consideration.

The sub-themes underwent further examination, refinement, and consolidation, resulting in the condensation of these sub-themes into three main themes. For each main theme, a detailed analysis of the codes was conducted, aiming to identify any additional sub-themes or subcategories that might represented distinct aspects or variations within that specific theme. This analytical process involved closely examining in an iterative process for patterns, differences, and additional layers of meaning, ultimately leading to the generation of new sub-themes and themes.

The codes were categorized multiple times into various categories until the final version of the themes were determined to appropriately capture the essence of the participants’ voices. This rigorous iterative approach enabled the researchers to identify significant patterns and themes within the data, leading to interpretations representing a comprehensive understanding of the participants’ experiences.

#### Trustworthiness

Trustworthiness is attained in a qualitative study when the findings of such a study are worth believing [19]. We applied the Guba and Lincoln criteria; credibility, dependability, transferability, and confirmability to enhance the trustworthiness of our study [20]. The credibility of this study’s findings was enhanced through transparent reporting, involvement of co-researchers, and building trust to encourage honest responses from the participants. In order to enhance the dependability of this study, we used the triangulation of researchers and data were collected using a semi-structured interview guide. The transferability of the findings of this study is enhanced through the description of the study context, data generation process and a transparent analytical approach. The researchers interpreted the dataset, and the findings reflect the participants’ perspectives, themes were both deductively and inductively generated from the interview transcript using thematic analysis approach. We also followed the Reflexive Thematic Analysis Reporting Guidelines (RTARG) for reporting as recommended by Braun and Clarke [21].

## Result

### Participants’ characteristics

A group of ten individuals were interviewed: eight males and two females. Their work experience had a median duration of six years (Table 1).

**Table 1.** Participant characteristics.

	Participant, n = 10	Participant (%)
**Gender**		
Male	8	80
Female	2	20
**Title/Role**		
Physicians and nurses	7	70
Others	3	30

### Theme one: feeling frustrated and stuck in the system: when care halts before it heals

**Figure 1.**
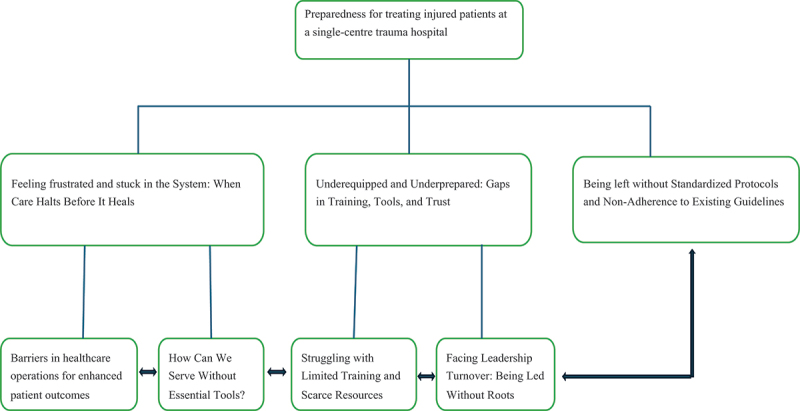
Themes and sub-themes of the analysis report.

The interviews shed light on challenges in personnel, medical supply handling, and facility management to streamline healthcare services to improve patient outcomes (Table 2 and Figure 1).

**Table 2.** Themes, sub-themes, and quote of the analysis report.

Themes	Sub-themes	Quote
Feeling Frustrated and Stuck in the System: When Care Halts Before It Heals	Barriers in Healthcare Operations for Enhanced Patient Outcomes	“A*fter resuscitation, a patient does not get a decision and does not move on to the next treatment or step. Here with us it is very slow to handover the patient from one treatment or medical procedure to next*.” (participant no.8)
	How Can We Serve Without Essential Tools?	“*The machines we are currently using are outdated, and the quality of the items is very poor. On top of this, most of the health professionals are unfamiliar with how to use them*.” (participant no.1)
Underequipped and Underprepared: Gaps in Training, Tools, and Trust	Struggling with Limited Training and Scarce Resources	“*Most health professional treating trauma patients here are not qualified in trauma care or emergency. Even if we try to save life and to resuscitate, we cannot provide quality treatment because there is no trauma surgeon*.” (participant no.9)
	Facing Leadership Turnover: Being Led Without Roots	*“Here it is just a changing of leader from one place/position to another, and this will prevent a lasting leadership.”* (participant no 2)
Being left without Standardized Protocols and Non-Adherence to Existing Guidelines		“*Am sorry I can’t recall any specific or general guideline. We just work as it should be. Only medical book guides us a standard.”* (participant no.6)

### Sub-theme 1a: barriers in healthcare operations for enhanced patient care

The interviews highlight significant challenges related to patient flow within ED, the intensive care unit (ICU) and orthopedic ward, as well as the absence of a dedicated rehabilitation center compounded by staff shortages.

Participants highlighted that the primary responsibility for patient care lies with resident doctors, which often leads to delays in decision-making. The senior physicians are not consistently present and are frequently in and out of the unit, leaving the residents to handle the treatment process. This lack of senior involvement hampers quick decision-making, as patients in need of urgent procedures have to wait for a senior physician’s input. In some cases, residents may lack the necessary skills or may be hesitant to make decisions without senior supervision.

The resident doctor holds his decision just not to be questioned by the senior or someone or she/he can see the patient case from the point of view of education only. (participant no.9)

The participants also highlight inconsistencies in provision of procedures. Procedures are frequently postponed, leading to a backlog of procedures and prolonged patient stays. There is no consistency in procedures availability or functionality. This lack of reliability makes it difficult to ascertain whether the services are actually accessible or not. Additionally, scheduled surgeries are frequently canceled for various reasons, such as unclean operating room attire, resulting in patients remaining untreated in the emergency room. The orthopedic injured patients in the ED, there is a significant number of patients waiting for procedures, but there is no senior decision-maker available to expedite the process. This leads to a slow pace of procedures and further delays. For patients waiting neurosurgery, procedures are performed relatively faster, but patients who require intensive care are sometimes brought back to the emergency room for ventilator use, instead of being transferred to the intensive care unit.

The interview highlights several challenges related to patient transfers. Although the initial reception and resuscitation of patients in the ED are relatively managed well, the process of transferring patients to definitive care poses a significant problem. Finding available beds in the intensive care unit or wards is difficult, resulting in patients staying in the emergency department longer than necessary. Some patients who undergo procedures in the operating room may not have access to beds in the ICU or recovery rooms, leading to their return to the emergency department and reliance on ventilators for support. This is particularly concerning for patients who have undergone head surgery. In addition, trauma patients who require rehabilitation after treatment are unable to be transferred to a suitable facility, resulting in their prolonged stay in the emergency department.

Receiving patients and providing first aid/resuscitation is in a good condition, but transferring patients to definitive care is a serious problem. It is always difficult to find a place to send the patient to the intensive care unit or ward. There is no bed so the patient will stay longer in the emergency department inappropriately. Patients after a procedure in operating room may not get bed in intensive care or recovery room, so the patient comes back to the emergency room and is put on a ventilator. (participant no.3)

The center faces constant bed shortages due to a low discharge rate, while there is an input of unidentified or unknown patients or homeless patients who cannot be transferred or sent home for recovery. Delayed decision-making further contributes to patients not being discharged from the emergency department promptly. Additionally, when patients are discharged from the ICU, the time required to clean and prepare beds for new admissions can be lengthy, further impeding the process of admitting new cases from emergency. Moreover, the orthopedic department’s delayed procedures result in patients waiting for extended periods, sometimes up to a week or more. Moreover, the total shortage of beds and inappropriate patient referrals from other health facilities contribute to congestion within the ED.

Relatedly, there is a lack of clear protocols or guidelines for patient reception and transfers between the ED and the ICU. The absence of admission criteria or guidelines for the ICU complicates the transfer process, leading to subjective decisions rather than standardized protocols. Similarly, there are no specific guidelines for transferring patients to the wards, relying on goodwill rather than established protocols.

The interviewee suggested several points for improving coordination and facilitating patient transfers for recovery or rehabilitation purposes. This includes finding appropriate transfer means and arranging external beds from other hospitals, which may involve communication and establishing rehabilitation services for post-trauma patients. Additionally, the interviewee suggests the importance of addressing the needs of patients who lack caregivers or those who are unidentified. Providing appropriate care and support for these individuals is essential to ensure their well-being and timely discharge from the ED. Furthermore, the interviewee highlights the potential benefit of opening and organizing semi-intensive care units. By creating such units, the patient load in the emergency department can be minimized, allowing for better management and care of patients in a more suitable environment.

Moreover, the interviews reveal a significant problem with manpower shortages, which is causing challenges in patient flow and adequate care. Many professionals are resigning, and the turnover rate seems to be high. The reasons cited include a lack of motivation, low salary, and the monotonous or tedious nature of working in the emergency room. These factors contribute to a stressful work environment.

The place we work is very stressful, but nothing is done to encourage or motivate the staff. (participant no.8)

As mentioned by the interviewee, to compensate for the shortage, extra time work is required, and additional compensation may be provided. However, these measures are temporary solutions and may not address the underlying issue of insufficient staff. The shortage of manpower is also attributed to the large number of patients requiring care. The high patient load further exacerbates the strain on the available staff members.

It is the big patient number that causes the shortage of manpower. I treated countless patients in a day. (participant no.5)

### Sub-theme 1b: how can we serve without essential tools?

The participants report about significant challenges related to the availability, maintenance, and procurement of medical equipment, as well as the shortage and inconsistent supply of medicines and materials. Some materials are in short supply or not available at all, leading to difficulties in providing adequate care. This includes shortages of essential items such as suction tubes, tracheostomy tubes, endotracheal tubes, intraosseous needle and suction machines. The lack of availability of equipment, including the C-arm machine and CT (computed tomography) scanner, hinders the delivery of necessary procedures and timely diagnostics. Moreover, the damage to equipment and machines is another pressing issue. resulting in patients having to wait or explore alternative options like private CT scans, which are often financially unaffordable. The damage predominantly stems from the negligent handling by professionals.

Machines always get damaged. For example, we have two C- arm machines, it always gets damaged and every procedure needs C- arm feezed. Same thing at CT. (participant no.10)

The procurement and distribution of medicines and equipment suffers from slow processes, bureaucracy, and a lack of a sustainable supply line. Delays in purchasing, importing, and distributing medicines and equipment result in shortages and stockouts. This not only affects patient care but also leads to financial burdens on patients who may have to purchase expensive items like screws themselves.

We are always faced with the loss of drugs or equipment from the market. We do not have a fixed and sustainable supply line. (participant no.7)

For management and appropriate handling of medical equipment, participants suggest consulting with the biomedical technician team to ensure completeness and evaluate the quality. Currently, purchases are often made without proper knowledge of the item or machine. By involving the biomedical technician team, they can assess the equipment thoroughly and ensure its functionality.

Secondly, they suggest a need for improvements in the availability and management of emergency drugs. It is recommended to proactively purchase drugs before they run out and monitor their expiration dates. Adequate quantities should be prepared based on the actual need to prevent wastage and expiration. Scientific assessment should be conducted to determine the appropriate amount to be procured. Additionally, addressing budget constraints is crucial to support these improvements. Lastly, they put an emphasis on working responsibly in the use of equipment. It is believed that if staff members handle and utilize the equipment properly, there will be a significant improvement in its reliability and longevity, reducing the frequency of failures and damages.

### Theme two: underequipped and underprepared: gaps in training, tools, and trust

This theme underscores a notable issue concerning the professional competencies of care providers, social service, biomedical technicians, and the trauma center leadership.

### Sub-theme 2a: struggling with limited training and scarce resources

The interviews highlight a significant knowledge and skill gap among healthcare professionals. It is noted that many professionals lack the necessary training and education in emergency/trauma management. The healthcare professionals treating trauma patients often rely solely on their experience rather than having formal qualifications in trauma care or emergency medicine. Furthermore, the lack of on-the-job training exacerbates this issue, as professionals are not being adequately prepared or updated in these critical areas, even those with a background in trauma or emergency education may still require up-to-date training to stay current with evolving practices. The problem is further compounded by the fact that new graduates are often hired without any specific training in emergency or trauma care, leading to a deficiency in their preparedness for the job. The knowledge and skills gap is evident even in crucial areas such as patient triage and the resuscitation zone. Coordination issues are also mentioned, indicating a lack of cohesive teamwork and efficient management.

Some professionals triage appropriately and if the patient needs help or treatment at triage, they take them to the resuscitation zone initiating the resuscitation. Some even don’t triage properly. (participant no.8)

Moreover, the absence of trauma surgery or qualified trauma surgeons is highlighted as a significant concern. This lack of understanding and presence of trauma surgery as a specialized field further contributes to the overall gap in trauma care.

It is highlighted that there is a lack of awareness among other experts regarding the role and scope of the social service department. Many staff members and experts are unaware of the specific problems that should be brought to the attention of social service workers. This lack of awareness and understanding of the department’s role creates a challenge in effectively addressing the social support needs of patients. Furthermore, it is mentioned that there is a problem with the attitude and perception towards social service workers, which state an issue with the trust to this service. ‘*They see us as a threat. They assume us, we are here to make the medical professionals to be accountable/questioned, but they don’t think we can help the patient.’* (participant no.2). This perception of social service workers as a threat rather than recognizing their role in protecting the safety and rights of patients, can stem from a fear of being held accountable or questioned by social service workers, which create a feeling of being accused, leading to a lack of cooperation and reluctance to engage with social service workers.

It is mentioned that biomedical technicians lack the necessary knowledge and skills required to effectively repair and maintain the equipment. This skill gap can result in improper or incomplete repairs, or even the failure to repair the equipment at all. On the other hand, it is noted that many health professionals have limited knowledge about operating the medical machines. They may lack the understanding necessary to effectively utilize and manipulate the machines. This lack of knowledge can lead to unintentional damage or misuse of the machines.

Additionally, there are instances where reports are received stating that a machine is not functioning properly, but upon investigation, no actual issue is found. This suggests a possible knowledge gap about the equipment from the health professionals’ side.

To enhance the skills and proficiency of the staff, participants made several recommendations. They emphasized the understanding that people, including healthcare professionals, are valuable assets and resources. The goodwill, sincerity, and dedication of the staff can make a significant difference in providing quality care despite limited resources.

It’s a poor country. We don’t have much, but we have to use what we have properly. (participant no.2)

While financial constraints may exist, it is recognized that factors beyond money, such as sincerity, play a pivotal role in bringing about positive change. Training and motivation are also identified as important aspects that can contribute to improving the trauma care system.

The interviewee expresses optimism that with the right approach, including utilizing available resources effectively, fostering goodwill, providing training, and promoting motivation among the staff can be achieved even in challenging circumstances. By recognizing and harnessing the human potential within the healthcare unit, and by fostering a sense of dedication and commitment, it is believed that improvements can be made, and quality care can be provided, regardless of the available limited resources.

### Sub-theme 2b: facing leadership turnover: being led without roots

The interviews shed light on the challenges related to leadership. Frequent changes in leadership positions occur frequently, without considering the specific leadership skills required for the role. This lack of stability and consistency in leadership positions can hinder the development of lasting leadership within the institution. However, it is also noted that leadership is currently improving, and efforts are being made to address issues and fix problems as they arise.

There is a frequent change of leaders but I can say leadership is not bad at all. (participant no.8)

The lack of accountability among practitioners is identified as a significant leadership issue. It is suggested that leaders should hold practitioners accountable for their work, ensuring they are responsible for the outcomes and quality of care they provide. Furthermore, the interviews indicate that training opportunities for staff members are not adequately facilitated or created by the management and leaders. Providing training is viewed as an important responsibility of the leadership team to enhance the skills and knowledge of the staff.

Overall, while there may be certain gaps in leadership, it is acknowledged that the overall leadership quality is reasonable, considering the high number of patients and resource scarcity.

### Theme three: being left without standardized protocols and non-adherence to existing guidelines

The interview clarifies that there is a substantial gap and significant challenges in quality of service and standardization.

The interviews reveal that the healthcare institution does not have a standardized trauma response team in place. Instead, tasks are assigned individually to different personnel without a structured team approach. It is mentioned that nurses or other healthcare professionals get assigned responsibilities for specific areas such as airway, breathing, or circulation during emergencies. However, there is no established trauma team that follows a standardized protocol for managing trauma cases. In situations where there is a high volume of danger or multiple victims, staff members from different units come together in a more ad hoc manner to address the situation. It is emphasized that there is no dedicated trauma team available, and specialists are only involved in patient care after consultation.

According to the interview, the ED has a positive and harmonious relationship among staff members. The professionals working together have a good rapport and there are no problems among colleagues. They collaborate well and maintain a positive working environment within the unit.

According to the interview, there might be situations where errors are sometimes suppressed. However, when a mistake does come to light, it is addressed through discussions, and attempts are made to solve the problem. The staff members gather together, have conversations, and collect reports in order to understand the issue and find solutions. They also report problems to their superiors.

The individuals involved in the error/mistake are spoken to, and the matter may be referred to an ethics committee for further investigation. Depending on the severity of the mistake, there may be disciplinary action or punishment. There is an approach of not ignoring problems and being proactive in addressing errors. They report errors, attempt to fix them within the unit if possible, and make a conscious effort to bring attention to the problems that arise. Furthermore, they are currently engaged in and actively working on improving the error analyzing situation. Regular checks for errors are being conducted, and there are plans for reviews in various sub-units to further enhance the quality of their work.

According to the interview, the trauma unit faces significant challenges due to the absence of a structured system and documentation. There is a lack of guidelines or protocols recognized by the whole departments for communication and collaboration. The guidelines that do exist are developed by individual departments, but they often face obstacles imposed by higher-level guidelines. This lack of a comprehensive and unified guide that incorporates research and discussions creates challenges in maintaining a standardized approach to treatment. The existing broken guidelines are not consistently implemented, leading to inconsistencies and potential rule violations.

One of the problems is that no one does follow the rules and regulations. I myself do not realize that there is a law to govern us as one. (participant no.5)

According to the interview, documentation is identified as a major gap. Nurses have historically not documented their work adequately, and efforts are being made to improve this aspect. However, not all Nurse professionals have implemented the changes yet.

The absence of clear infection prevention protocols and guidelines is highlighted as a significant gap. There is a lack of a consistent and managed infection control system, and different individuals may have different approaches to preventing infections.

If you ask five people, all five will tell you that there are different ways to prevent infection. We do not have a single managed and controlled infection control system. (participant no.9)

The overall system of the hospital is described as lacking proper standardization. Protocols established by departments are sometimes broken due to inconvenience or practicality issues.

Based on the responses from the interviewees, it can be inferred that the overall assessment of the service quality is mixed. While some interviewees consider it is fair and acknowledge the challenges of serving a large population, others express dissatisfaction and believe that more improvements are needed. There is a consensus that there are gaps in the service and that more needs to be done to meet the standard. The lack of space and resources is mentioned as a contributing factor to the service limitations. Despite these challenges, it is noted that the service is not very bad but still falls below expectations.

One interviewee suggests that the service quality is low and complicated. They indicate that they have observed recurring problems and have come to consider them normal or expected.

I can even say the quality is low, it’s complicated. I always looks the same problem until I consider it normal or say it’s a known problem. (participant no.4)

The feedback received from the interviewees indicates that patient satisfaction is not consistent, with mixed experiences and opinions expressed. It is acknowledged that some patients come out almost happy, while others experience dissatisfaction. Based on the interviewees, one common concern raised by patients is the significant waiting time before undergoing procedures. Patients often have to wait for extended periods, sometimes without even getting a bed and having to sit on a chair instead.

Patients may even sit on the chair without getting a bed. There are times when the patient often complains, and there are times when they are happy. They wait a lot without having the procedure done for them. (participant no.3)

Complaints about procedure delays and a lack of beds are frequently mentioned by patients. In some cases, patients have reported stays of up to two months in the emergency department, and even if procedures are expedited, there can be a waiting period of two weeks or more. The delay in decision-making and the uncertainty surrounding whether procedures will be conducted or if patients will be transferred to the ward add to the difficulties faced by patients.

The prolonged duration of their stay leads to frustration and dissatisfaction. This extended waiting time appears to be a significant issue that negatively impacts the overall patient experience.

## Discussion

In this study, we gained insight into the perspectives of staff regarding the preparedness of their trauma center for treating injured patients. The participants’ insights focused on patient flow, professional competencies, resource allocation, and standardization with our discussion advocating for improvements in trauma care delivery.

In critical trauma situations, timely decision-making is essential to patient outcomes [22]. However, our finding that resident doctors bear the primary responsibility for patient care, often without the immediate presence of senior physicians for decision, suggests potential delays in patient care. This also contributes to residents feeling inadequately supported, which in turn affects their confidence and decision-making capacity. The findings also highlight inconsistencies in the availability and functionality of procedures, leading to delays and backlogs that negatively affect patient outcomes. The absence of senior decision-makers, particularly for the orthopedic injured patients, appears to contribute to the slow pace of procedures. Research has demonstrated that inconsistent access to essential procedures can adversely impact patient flow and outcomes [23]. These delays create a bottleneck effect, disrupting patient flow throughout the hospital as patients remain in emergency or other holding areas while awaiting treatment [24].

Delays in transferring patients from the ED to definitive care, particularly to ICU or ward beds, can compromise patient outcomes and exacerbate ED overcrowding. Overcrowding in the ED is a well-documented global issue in healthcare and is often associated with increased patient wait times and reduced care quality [25,26]. These challenges in securing ICU or ward beds reflect resource constraints that can limit the quality and timeliness of care for patients requiring intensive monitoring and treatment. Prolonged ED stays due to transfer delays also hinder the ED’s ability to effectively triage and treat new incoming patients, negatively impacting overall department functionality. As the ED is often the first point of contact for acute and emergency care, optimizing patient flow is essential to maintaining its capacity to handle emergencies efficiently [27].

Similarly, the lack of essential equipment, such as C-arm x-rays machines and CT scanners, leads to delays in necessary diagnostics and procedures, forcing patients to wait or seek alternatives outside the hospital. Delayed diagnostics can result in missed or postponed treatment, potentially worsening patient outcomes [28]. Additionally, the shortage of critical medical supplies and equipment, such as suction devices and tracheostomy tubes, directly impacts the quality of patient care. Such shortages hinder healthcare providers’ ability to perform vital procedures, which can lead to suboptimal outcomes [29]. The inconsistent availability of essential equipment and supplies ultimately affects the overall quality and efficiency of care by straining healthcare providers, lowering the standard of care, and extending patient wait times [29].

Trauma and emergency medicine require specialized skills and knowledge, including rapid assessment, decision-making, and life-saving interventions, which are difficult to acquire solely through experience [30]. However, our study revealed a concerning trend of inadequate formal training and education in emergency and trauma care among healthcare professionals. Without proper training, staff may feel unprepared to handle complex or high-pressure situations, potentially compromising patient outcomes. Furthermore, while continuous training is essential in emergency care, where protocols and best practices evolve rapidly [31], there is a lack of structured, on-the-job training, which further limits healthcare workers’ ability to manage trauma cases effectively. Resource constraints, lack of awareness, and insufficient policy support could be contributing factors [32].

Research shows that hospitals with dedicated trauma teams typically achieve better patient outcomes, as these teams follow established protocols that streamline decision-making and interventions during emergencies [33]. Our study revealed a lack of a standardized trauma response team, highlighting a significant gap in the institution’s approach to trauma care. A structured trauma team, often composed of a designated group of professionals specifically trained to respond to trauma cases, ensures a cohesive and systematic approach that is crucial for rapid and effective responses in life-threatening situations [34]. Assigning tasks individually and relying on ad hoc teamwork can lead to communication breakdowns, role confusion, and delayed responses [34]. In trauma care, where every second counts, the absence of a cohesive team structure can result in inefficient workflows and missed steps in patient assessment and treatment.

Furthermore, in mass casualty situations, an unstructured response can lead to overcrowding, poor resource allocation, and potentially dangerous environments for both patients and healthcare workers [35]. Standardized trauma teams with clear roles and protocols are essential for managing such incidents effectively, minimizing chaos, and ensuring an organized response. The challenges in organizing trauma teams stem from various factors, including the absence of standardized trauma team activation protocols, resource constraints such as limited trauma team members, inefficient communication systems, inadequate training and education, workflow complexities, and hierarchical workplace cultures that prioritize authority over teamwork and collaboration [36,37].

The absence of a standardized trauma-specific guideline recognized across all departments creates significant issues for patient care. This also highlights a gap in communication and collaboration between departments. When each department operates based on its own protocols, it can lead to fragmentation in treatment approaches and inconsistencies that may compromise patient outcomes [38]. Like other healthcare services, trauma care requires coordinated, interdisciplinary actions, and fragmented guidelines hinder this essential collaboration [38].

Consistent application of guidelines in trauma care reduces variability in treatment, improves patient outcomes, and enhances the overall quality of care [39,40]. However, our study indicated that existing guidelines, even when present, are reportedly not consistently implemented across the trauma unit, could contribute to variability in care. A culture of units operating semi-independently, prioritizing different aspects of trauma care, resource constraints in developing and implementing comprehensive guidelines, unawareness of existing guidelines, and fear of legal or professional repercussions if guidelines are misapplied are described to contribute to lack of establishing standardized guidelines and inconsistent implementation [41–43].

Furthermore, staff shortages and high patient volumes place immense pressure on healthcare workers, contributing to burnout, emotional exhaustion, and increased turnover [44]. Our study further underscores this issue, revealing significant levels of burnout and staff turnover. As experienced personnel leave, they are often replaced by less seasoned staff who may struggle with the complexities of trauma care and established protocols. This diminishes the overall quality of care and increases the burden on remaining staff, perpetuating a cycle of turnover closely linked to declining patient outcomes and higher mortality risks for patients [44,45].

### Future direction

Our findings provide valuable insights that contribute to hospital-based improvements, enhancing the quality of care and optimizing patient outcomes [46]. A rotational system instead of on-call to ensure senior physicians available for decision-making during critical trauma situations should be implemented. Introducing an intermediate care unit (semi-ICU) as a bridge for patients who no longer require ICU-level care but are not yet ready for regular ward transfer would be beneficial [44]. Establishing clear protocols for prioritizing ICU or ward transfers for critically injured patients would be useful. Furthermore, to improve ED efficiency, measures such as expediting discharges through early discharge planning, ensuring outpatient follow-up, and conducting regular audits to identify bottlenecks in patient transfer processes and resolve inefficiencies are necessary. Introducing formal, simulation-based, and competency-based training programs for all staff working in trauma settings will enhance professional development [47,48]. Establishing dedicated trauma response teams composed of multidisciplinary professionals, along with trauma team activation protocols, is feasible and fundamental for improving patient outcomes [33]. Overall, in the context of Global South countries, the implementation of quality improvement initiatives and structural system enhancements is critical to reducing mortality rates and optimizing outcomes within trauma care systems [49].

### Methodological consideration

This study has several strengths, but also some limitations. Firstly, to the best of our knowledge, this is the first study to assess participants’ perspectives in this specific trauma care setting in a LMIC country serving a large population. Secondly, the sample pool included a diverse group of professionals working in various roles and departments, ensuring a range of perspectives and experiences were represented. Thirdly, the dataset generation and analysis process were transparent, with themes inductively generated from the interview transcripts using a thematic analysis approach. Lastly, the analysis was collaboratively shaped and refined across all phases with co-researchers, enhancing reflexivity and increasing the reliability of the findings.

A smaller sample size was one of the limitations of our study. However, we used a strategic sampling approach by selecting participants who could provide rich and relevant insights, rather than aiming for statistical representativeness. This approach allowed for in-depth analysis of each participant’s perspective and was guided by the concept of data saturation [17]. Additionally, a smaller sample made the processes of conducting, transcribing, and analyzing the data more manageable while maintaining high quality.

Subjectivity and bias were also anticipated limitations in our study. However, we made a concerted effort to mitigate these potential biases during both the interviews and the analysis. While themes are inherently based on researchers’ interpretations, which may introduce bias or reflect preconceived notions, we ensured that themes were inductively generated to maintain rigor and reflect the participants’ voices. Our findings may be context specific and based on a representative sample, but they provide a strong depiction of the primary trauma center in the country. Moreover, the findings are applicable to broader populations in similar settings.

## Conclusion

The study highlights several critical challenges impacting the delivery of patient care. There are significant gaps in terms of hospital preparedness for caring for injured patients, and these lacks can lead to delays in treatment and poor clinical outcomes. Resident doctors, who bear the primary responsibility for patient care, often face delays in decision-making, which contribute to procedural backlogs and extended patient stays. Issues related to patient transfers further exacerbate these delays. Additionally, significant challenges were identified in the availability, maintenance, and procurement of medical equipment, alongside shortages and inconsistencies in the supply of medicines and materials. A notable knowledge and skill gap among healthcare professionals was observed, compounded by the absence of a standardized trauma response team. Furthermore, the institution lacks structured guidelines and protocols.

## Supplementary Material

Interview guide.docx

## Data Availability

The datasets used and/or analysed during the current study are available from the corresponding author on reasonable request.

## References

[1] Reynolds TA, Stewart B, Drewett I, et al. The impact of trauma care systems in low-and middle-income countries. Annu Rev Public Health. 2017; 38:507–13. doi: 10.1146/annurev-publhealth-032315-021412 28125389

[2] Debas HT, Donkor P, Gawande A, et al. Essential surgery: key messages from disease control priorities, 3rd edition. Lancet. 2015;385:2209–2219. doi: 10.1016/S0140-6736(15)60091-5 25662414 PMC7004823

[3] World health organization. Injuries and violence facts. 2021 [cited 2024 Oct 19]. Available from: https://www.who.int/news-room/fact-sheets/detail/injuries-and-violence

[4] World Bank. Proportion of deaths by injury. 2019 [cited 2024 Dec 17]. Available from: https://data.worldbank.org/indicator/SH.DTH.INJR.ZS?end=2019&start=2019&view=map

[5] United nations: African renewal. Road safety week: African nations steer towards reducing deaths. 2023 [cited 2024 Oct 19]. Available from: https://www.un.org/africarenewal/magazine/may-2023/road-safety-week-african-nations-steer-towards-reducing-deaths

[6] Mock C, Joshipura M, Arreola-Risa C, et al. An estimate of the number of lives that could be saved through improvements in trauma care globally. World J Surg. 2012; 36:959–963. doi: 10.1007/s00268-012-1459-6 22419411

[7] World Health Organization. Guidelines for essential trauma care. World Health Organization; 2012 [cited 2023 Mar 17]. Available from: https://www.who.int/publications/i/item/guidelines-for-essential-trauma-care

[8] MacKenzie EJ, Rivara FP, Jurkovich GJ, et al. A national evaluation of the effect of trauma-center care on mortality. N Engl J Med. 2006;354:366–378. doi: 10.1056/NEJMsa052049 16436768

[9] Choi J, Vendrow EB, Moor M, et al. Development and validation of a model to quantify injury severity in real time. JAMA Netw Open. 2023; 6:e2336196. doi: 10.1001/jamanetworkopen.2023.36196 37812422 PMC10562944

[10] Callese TE, Richards CT, Shaw P, et al. Trauma system development in low-and middle-income countries: a review. J Surg Res. 2015; 193:300–307. doi: 10.1016/j.jss.2014.09.019 25450600

[11] World Health Organization. Rehabilitation [Internet]. Geneva: World Health Organization; 2024 [cited 2025 Apr 14]. Available from: https://www.who.int/news-room/fact-sheets/detail/rehabilitation

[12] El Sayed MJ. Developing emergency and trauma systems internationally: what is really needed for better outcomes? J Emerg Trauma Shock. 2017; 10:91–92. doi: 10.4103/JETS.JETS_63_16 28855768 PMC5566039

[13] Kruk ME, Gage AD, Arsenault C, et al. High-quality health systems in the sustainable development goals era: time for a revolution. Lancet Glob Health. 2018; 6:e1196–252. doi: 10.1016/S2214-109X(18)30386-3 30196093 PMC7734391

[14] Ali S, Destaw Z, Misganaw A, et al. The burden of injuries in Ethiopia from 1990–2017: evidence from the global burden of disease study. Inj Epidemiol. 2020; 7:1. doi: 10.1186/s40621-020-00247-0 33342441 PMC7751094

[15] Wallis LA, Gottschalk SB, Wood D, et al. The Cape triage score—a triage system for South Africa. S Afr Med J. 2006; 96:53–56. 16440113

[16] Malterud K, Siersma VD, Guassora AD. Sample size in qualitative interview studies: guided by information power. Qual Health Res. 2016; 26:1753–1760. doi: 10.1177/1049732315617444 26613970

[17] Hennink M, Kaiser BN. Sample sizes for saturation in qualitative research: a systematic review of empirical tests. Soc Sci Med. 2022;292:114523. doi: 10.1016/j.socscimed.2021.114523 34785096

[18] Braun V, Clarke V. Reflecting on reflexive thematic analysis. Qual Res Sport Exerc Health. 2019; 11:589–597.

[19] Graneheim UH, Lundman B. Qualitative content analysis in nursing research: concepts, procedures and measures to achieve trustworthiness. Nurs Educ Today. 2004; 24:105–112. doi: 10.1016/j.nedt.2003.10.001 14769454

[20] Guba EG, Lincoln YS. Guidelines and checklist for constructivist (aka fourth generation) evaluation. Thousand Oaks (CA): Sage Publications; 2001 Nov.

[21] Braun V, Clarke V. Supporting best practice in reflexive thematic analysis reporting in palliative medicine: a review of published research and introduction to the reflexive thematic analysis reporting guidelines (RTARG). Palliat Med. 2024; 38:608–616. doi: 10.1177/02692163241234800 38469804 PMC11157981

[22] Madani A, Gips A, Razek T, et al. Defining and measuring decision-making for the management of trauma patients. J Surg Educ. 2018; 75:358–369. doi: 10.1016/j.jsurg.2017.07.012 28756147

[23] Raeisi A, Rarani MA, Soltani F. Challenges of patient handover process in healthcare services: a systematic review. J Educ Health Promot. 2019; 8:173. doi: 10.4103/jehp.jehp_460_18 31867358 PMC6796291

[24] Robinson B, Purcell LN, Reiss R, et al. Reasons for in-hospital delays to emergency surgical care in a resource-limited setting: surgery versus anesthesiology perspective. Trop Doct. 2023; 53:66–72. doi: 10.1177/00494755221100342 35892158

[25] Sartini M, Carbone A, Demartini A, et al. Overcrowding in emergency department: causes, consequences, and solutions—a narrative review. Healthcare. 2022; 10:1625. doi: 10.3390/healthcare10091625 36141237 PMC9498666

[26] Pearce S, Marchand T, Shannon T. Emergency department crowding: an overview of reviews describing measures, causes, and harms. Intern Emerg Med. 2023; 18:1137–1158. doi: 10.1007/s11739-023-03085-6 36854999 PMC9974385

[27] Samadbeik M, Staib A, Boyle J, et al. Patient flow in emergency departments: a comprehensive umbrella review of solutions and challenges across the health system. BMC Health Serv Res. 2024; 24:274. doi: 10.1186/s12913-024-10725-6 38443894 PMC10913567

[28] Balogh EP, Miller BT, Ball JR. National academies of sciences, engineering, and medicine. Overview of diagnostic error in health care. In: Balogh EP, Miller BT, Ball JR, editors. Improving diagnosis in health care. Washington (DC): National Academies Press (US); 2015. p. 81–144.

[29] Zamzam AH, Abdul Wahab AK, Azizan MM, et al. A systematic review of medical equipment reliability assessment in improving the quality of healthcare services. Front Public Health. 2021; 9:753951. doi: 10.3389/fpubh.2021.753951 34646808 PMC8503610

[30] Awwad K, Ng YG, Lee K, et al. Advanced trauma life support/advanced trauma care for nurses: a systematic review concerning the knowledge and skills of emergency nurses related to trauma triage in a community. Int Emerg Nurs. 2021; 56:100994. doi: 10.1016/j.ienj.2020.100994 33798982

[31] Omukeeva G, Shermatova U, Dushimbekova K, et al. Analysis of continuous training effectiveness of doctors and nurses in emergency. Pak J Med Health Sci. 2021;15:3647–3652. doi: 10.53350/pjmhs2115123647

[32] Nyamtema A, Karuguru GM, Mwangomale AS, et al. Factors affecting production of competent health workforce in Tanzanian health training institutions: a cross-sectional study. BMC Med Educ. 2022; 22:662. doi: 10.1186/s12909-022-03918-0 36064387 PMC9446711

[33] Nurlaelah S, Kamal AF, Irawati D, et al. Trauma team activation in the emergency department: a literature review of criteria, processes and outcomes. Malays J Med Health Sci. 2024; 20:323–329. doi: 10.47836/mjmhs.20.1.40

[34] Chien DS, Yiang GT, Liu CY, et al. Association of in-hospital mortality and trauma team activation: a 10-year study. Diagnostics. 2022; 12:2334. doi: 10.3390/diagnostics12102334 36292022 PMC9600103

[35] DeNolf RL, Kahwaji CI. EMS mass casualty management. Eur PMC. 2018; 24:66. 29493995

[36] Cooper BH. Exploring the factors that influence trauma team activation in emergency department staff. Emerg Nurse. 2022; 30:25–32. doi: 10.7748/en.2022.e2133 35502574

[37] Hardcastle TC, Oteng R. Trauma care in Africa: triumphs and challenges. Afr J Emerg Med. 2011;2:53–54. doi: 10.1016/j.afjem.2011.07.002

[38] Ebben RH, Siqeca F, Madsen UR, et al. Effectiveness of implementation strategies for the improvement of guideline and protocol adherence in emergency care: a systematic review. BMJ Open. 2018; 8:e017572. doi: 10.1136/bmjopen-2017-017572 PMC6254419 30478101

[39] Beauchemin M, Cohn E, Shelton RC. Implementation of clinical practice guidelines in the health care setting: a concept analysis. Adv Nurs Sci. 2019; 42:307–324. doi: 10.1097/ANS.0000000000000274 PMC6717691 30839334

[40] Alharbi RJ, Shrestha S, Lewis V, et al. The effectiveness of trauma care systems at different stages of development in reducing mortality: a systematic review and meta-analysis. World J Emerg Surg. 2021; 16:1–2. doi: 10.1186/s13017-021-00346-4 34256793 PMC8278750

[41] Mansoor T, Puteh SE, Aizuddin AN, et al. Challenges and strategies in implementing hospital accreditation standards among healthcare professionals in healthcare systems in Yemen: a phenomenological study. Cureus. 2024; 16. doi: 10.7759/cureus.59383 PMC11139055 38817454

[42] Zarifraftar M, Aryankhesal A. Challenges of implementation of accreditation standards for health care systems and organizations: a systematic review. J Manag Sci. 2016;2:191–201.

[43] Tian C, Xu M, Wang Y, et al. Barriers and strategies of clinical practice guideline implementation in China: aggregated analysis of 16 cross-sectional surveys. J Public Health. 2024; 32:1891–1904. doi: 10.1007/s10389-024-01556-4

[44] Shen K, McGarry BE, Gandhi AD. Health care staff turnover and quality of care at nursing homes. JAMA Intern Med. 2023; 183:1247–1254. doi: 10.1001/jamainternmed.2023.5225 37812410 PMC10562988

[45] Moscelli G, Mello M, Sayli M, et al. Nurse and doctor turnover and patient outcomes in NHS acute trusts in England: retrospective longitudinal study. BMJ. 2024; 387. doi: 10.1136/bmj-2024-079987 PMC11577445 39566973

[46] Kistler EA, Klatt E, Raffa JD, et al. Creation and expansion of a mixed patient intermediate care unit to improve ICU capacity. Crit Care Explor. 2023; 5:e0994. doi: 10.1097/CCE.0000000000000994 37868027 PMC10586855

[47] Elendu C, Amaechi DC, Okatta AU, et al. The impact of simulation-based training in medical education: a review. Medicine (baltimore). 2024; 103:e38813. doi: 10.1097/MD.0000000000038813 38968472 PMC11224887

[48] Rowe AK, Rowe SY, Peters DH, et al. The effectiveness of training strategies to improve healthcare provider practices in low-income and middle-income countries. BMJ Glob Health. 2021; 6:e003229. doi: 10.1136/bmjgh-2020-003229 PMC7813291 33452138

[49] Jin J, Akau’ola S, Yip CH, et al. Effectiveness of quality improvement processes, interventions, and structure in trauma systems in low- and middle-income countries: a systematic review and meta-analysis. World J Surg. 2021; 45:1982–1998. doi: 10.1007/s00268-021-06065-9 Epub 2021 Apr 9. PMID: 33835217. 33835217

