# Supplementary material for: Preparedness for treating injured patients at a single-centre trauma hospital in Ethiopia: a qualitative study
Source: Glob Health Action. 2025 Aug 5;18(1):2540669. doi: 10.1080/16549716.2025.2540669 (PMC12326381; doi:10.1080/16549716.2025.2540669)
Supplement: Interview guide.docx [file ZGHA_A_2540669_SM1122.docx]

**Interview guide questions: Preparedness for treating injured patients at a single-center trauma hospital in Ethiopia: a qualitative study**

1. What are your overall perceptions and experiences of the quality of trauma care/service provided at your trauma center?
2. To what extent do you think that the patients at your trauma center is receiving the best care/service? Why? Please elaborate. Suggestion: What are your challenges in your daily work?
   1. Possible follow-up:
      1. Leadership?
      2. Colleagues?
      3. Human resources in general?
      4. System? Documentation?
      5. Equipment?
      6. Patient satisfaction?
      7. Next of kin/caregivers?
3. How do you manage your challenges?
   1. Possible follow-up question: In your experience, what are important efforts or adjustments in the daily work to ensure the patient safety for the injured patients?
4. What are your experiences with the hospitals work with analyzing errors in the emergency care?
5. Can you describe your experiences you might have faced in your trauma center regarding the work with protocol or guidelines for the emergency care work?
6. (not applicable question for biomedical engineer and pharmacist). Can you describe and elaborate on your experience and challenges on the patient flow starting from:
7. Patient triage
8. Resuscitation zones
9. Patient transfer
10. Can you describe and elaborate on your experiences related to how the trauma teamwork at your trauma center is in terms of:
11. Team activation
12. Involved group of professionals
13. Communication
14. Can you describe your experiences and challenges on accessing various disciplinary units necessary for the care of the injured patient?
15. What do you think can improve the quality of care in your trauma center? Please elaborate on what you think is the most important aspect regarding your experiences.
